# Pragmatic skills in people with Williams syndrome: the perception of families

**DOI:** 10.1186/s13023-024-03016-0

**Published:** 2024-03-01

**Authors:** Esther Moraleda Sepúlveda, Patricia López Resa

**Affiliations:** Faculty of Psychology, University Complutense of Madrid, Campus de Somosaguas, 28223 Pozuelo de Alarcón, Spain

**Keywords:** Pragmatics, Williams syndrome, Language, Parents, Family perception

## Abstract

**Background:**

One of the most challenging linguistic areas in people with Williams Syndrome throughout their evolutionary stage is the development of pragmatic skills. The research conducted so far highlights specific problems concerning adaptation to the linguistic context and interlocutors, language comprehension, as well as other aspects interfering with verbal communication. However, until now, most scientific evidence has been based on personal assessments of this group. In a complementary manner, the goal of this study was to discover the level of pragmatic skills of people with Williams Syndrome from the point of view of the families. The sample consisted of 34 families belonging to the Williams Syndrome Association of Spain. The assessment instrument was the pragmatic awareness questionnaire, which includes 26 items related to different aspects that are part of the pragmatic area on a Likert-type scale.

**Results:**

The results indicate that, families consider there to be a regular to low level in all the areas assessed. The strong points seem to lie in the paralinguistic aspects, while the weakest factors are those related to the understanding of figurative language**.**

**Conclusions:**

Therefore, it is necessary to continue insisting on the importance of language intervention in this group throughout its development to improve its level of linguistic competence.

**Supplementary Information:**

The online version contains supplementary material available at 10.1186/s13023-024-03016-0.

## Introduction

Williams Syndrome (hereinafter ‘WS’) is a genetic disorder caused by a deletion in chromosome 7q11.23 [76]. WS is estimated to affect 1 in 20,000/50,000 live births worldwide [49].

The first studies conducted with this population began to show an atypical neuropsychological profile characterized by peaks and valleys in the various abilities [25]. For example, Bellugi et al. [7] showed that abilities such as language was well preserved in contrast to other abilities such as visuospatial construction cognition, attention, or visuospatial memory [40]. Subsequent works have confirmed that, although it is true that people with WS exhibit a series of characteristic features, there is a profile that is incredibly open to individual variability [43].

Although the clinical profile of this population has led some authors to interpret WS as an argument evidencing the feasibility of the theory of modular language functioning [60], there is currently much research suggesting that the dissociation between linguistic production and cognitive functioning does not exist in people with WS. For this reason, when analyzing these abilities in-depth, specific difficulties of people with WS become apparent in the different linguistic areas [53]. For example, at the phonetic-phonological level, there is unintelligible articulation, phonological paraphasia and a general delay in acquisition manifested through various phonological simplification processes—such as assimilation or omission, malapropism, and epenthesis—persisting beyond the expected norm [25]. However, it is also interesting to note that both phonological segmentation tasks and word and pseudoword repetition tasks can lead to scores that are very similar to those of subjects with typical development [20, 37, 57, 74].

Concerning the morpho-syntactic level, people with WS again seem to manifest profiles below those corresponding to typical development [24]. For example, difficulties in the correct use of the morphemes of time, gender and number are particularly noteworthy,and even the understanding, production, and repetition of structurally complex utterances [24, 55, 58].

Regarding the lexical-semantic level, it has been observed that there are several differences in this area in people with WS. Although some strong points are lexical decision tasks and semantic association, they experience many more problems in other tasks such as naming pictures, manifesting specific difficulties in word retrieval, possibly because of lesser sustained attention [24]. For their part, Mervis and Becerra [39] reported a relative preservation of the lexical component in people with WS and Garayzábal and Cuetos [20] evidenced the existence of significant differences between the relational and conceptual lexicon. Along this line, the study conducted by Moraleda and López [43] revealed how, although people with WS obtain scores below what one might expect based on their mental age, the passive lexicon of this population increases in proportion to their chronological age.

In the realm of pragmatics and language usage, there exists notable divergence among various research studies. While less recent research assures us that people with WS do not experience problems in this area [44, 50, 75], other studies show that this area is delayed when compared, for example, to the development of the semantic area [28], Van den [69].

Specifically with regard to the pragmatic characteristics of people with WS, there has been evidence of poor turn-taking skills [4, 69], difficulties with understanding figurative language [71], excessive use of emotional language [70] and limited use of textual markers in narrative construction [13], among other aspects. More recent studies indicate that in many cases, WS is accompanied by a Social Communication disorder [14], following previous studies that associated WS with social communication deficits, in the form of atypical social interaction (conversational exchange structure, turn-taking and information transfer) and conversational insufficiency [59].

For their part, authors such as Gallardo [18], Lacroix et al. [33] and Royston et al. [54] have shown the existence of a specific deficit in the Gricean principle of cooperation [23] manifesting itself through a series of alterations in conversational maxims. Specifically, it has been observed that, although the linking of conversational turns occurs smoothly [32], constant overlapping [16] makes it difficult to understand the relevance of the interlocutors’ contributions (maxim of relation). This, in turn, makes it difficult to take adequate reactive turns [72]. In this sense, Aroca and Liras [10] and Diez-Itza et al. [13] also attribute the continuous changes in discursive themes to difficulties with the maxim of relation, which is crucial to determining when a contribution is relevant to the communicative situation. Likewise, various research studies have found that some of the lexical-semantic peculiarities of WS, such as the choice of atypical and infrequent words [38], can be explained by difficulties with the maxim of manner, which is necessary for adapting language to the communicative context [54, 66]. In a similar vein, certain authors associate the characteristic language patterns of individuals with Williams syndrome (WS) with challenges in managing information overload. This implies that individuals with WS may struggle to discern instances of excessive speech or situations where insufficient information is conveyed. [3].

There are also many research studies addressing the study of the theory of mind in people with WS [6, 30, 54, 56, 73].

Thus, some authors suggest that people with WS experience a dissociation between their social and cognitive profiles in terms of mentalizing skills (understood as the ability to make inferences about the thoughts and beliefs of other people), although the evidence is contradictory. Initial research suggested that these abilities were preserved in WS, unlike other cognitive abilities [31]. However, Tager-Flusberg and Sullivan [64] demonstrated that the socio-perceptual components of the theory of mind, such as facial expression, body language or vocal prosody would be preserved while the socio-cognitive components such as language and understanding of false beliefs [63] would be impaired [45]. However, Porter et al. [47] showed that theory of mind skills in people with WS are below what would be expected for their mental age when they are assessed using a non-verbal task, not being able to depend on their verbal skills. Therefore, it seems difficult to reach a clear conclusion about mentalizing abilities in people with WS, although their potential difficulties in this domain are more closely related to the social-cognitive component than to the social-perceptive one [67].

All this information suggests the existence of a specific difficulty in the assessed pragmatic skills of people with WS. However, there is little scientific literature on the perception of parents concerning these specific difficulties. Bearing in mind that it is families that spend the most time with them in natural contexts and given the importance of being direct observers [42], the goal of this study was to determine the pragmatic characteristics of people with WS from the point of view of their families.

## Method

### Participants

The sample comprises a total of 34 families with children diagnosed with WS, aged between 8 and 20 years, 52.9% female and 47.1% male, residing in Spain. Specifically, 26 fathers between the ages of 32 and 65 and 18 mothers between the ages of 29 and 59 participated in this study.

It is noteworthy that all people with WS presented a certificate of disability equal to or greater than 33%. A degree of disability of 33% indicates a disability that prevents you from conducting certain activities but does not prevent you from functioning in your current environment. It is regulated by the Spanish public administration and is issued by the Assessment and Orientation Teams made up of at least a doctor, psychologist, and social worker. About people with WS, it should be noted that 50% were attended by a speech and language therapist. The participants belong to the Williams Syndrome Association of Spain (ASWE). They all speak Spanish as their first language.

### Instruments

To conduct this study, the pragmatic awareness questionnaire (PAQ) [52] was distributed to the families of people with WS. This questionnaire follows the integrative line of the ‘PerLa’ corpus [17] of clinical data on aphasia and evaluation protocols such as the Rapid Pragmatic Evaluation Protocol—Revised (PREP-R) [15] and draws inspiration from the Pragmatic Protocol Manual by Prutting and Kirchner [48], from which the categories that most closely resemble the WS profile in the PAQ have been selected and introduced (Additional file 1). This questionnaire had already been used previously in other studies in the population with intellectual disabilities [9, 52].

The PAQ is made up of blocks, divided into 26 items that are distributed as ordinal variables, following a 5-point Likert-type scale, where 1 = very bad, 2 = bad, 3 = fair, 4 = good and 5 = very good. The wording of some items has been simplified and examples have been added for ease of understanding.

Each block of the questionnaire pertains to a different area. In block I (items 1–2), intelligibility (effectiveness in understanding spoken voice) and paralanguage (intonation, volume, etc.) are assessed. In block II (items 3–8), aspects of non-verbal communication are measured, such as the physical contact maintained during the communicative act or the distance established between the interlocutors, along with factors such as body posture, gaze, facial expression, and the use of gestures. Block III (items 9–10) focuses on lexical competence and cohesion. Block IV (items 11–13) assesses semantic-pragmatic capacities, related to the understanding of irony and humor, and the interpretation of ambiguous statements. Block V (items 14–16) refers to the morpho-syntactic characteristics of the discourse. In block VI (item 17), the ability to adapt to the interlocutor and the communicative situation is assessed. Lastly, block VII (items 18–25) focuses on the amount and relevance of information in communicative exchanges, response time, control over turn-taking, the degree of acceptance and coherence of the topics put forward by the subject and the ability to follow the conversation. The last item (26) is an open qualitative question concerning general communication, where the perception and concerns that families of people with WS may have about their level of pragmatic skills are collected in writing.

The criteria to measure reliability were: (a) Cronbach’s alpha, which allowed obtaining a value of 0.972; (b) the standardized alpha (0.969), and (c) the correlation of each item with the total of the scale (item-total correlation), which for all the items exceed the value 0.20.

### Procedure

First, the Williams Syndrome Association of Spain—the center from which the participants were selected—was contacted. A document was drawn up with a brief description of the study, its goals, the pertinent instructions, and the importance of family participation. Likewise, the document contains a Google Forms link to the questionnaire that was used, where the informed consent also appears. After approval by the Association and acceptance by the Ethics Committee of the University’s Faculty of Health Sciences, the families were given the document containing the questionnaire to be filled out. The approximate time needed to answer all the questions was 15 min.

Once all the questionnaires were completed, the data collected was subjected to a descriptive statistical analysis for the purposes of studying parents’ perception of their children’s level of pragmatic skills and the characteristics thereof. For this research, statistical analysis was performed using SPSS 24.0. The most important descriptive statistics were obtained. To analyze the correlation between variables, Spearman’s Rho test was used.

## Results

The results obtained have shown that, from the parents’ perspective, people with WS experience difficulties in the various items of pragmatics. The general averages of the results can be seen in Table 1.

**Table 1:** Mean scores in the sections of the pragmatic awareness questionnaire

Items assessed	Average score	Range score
1. Intelligibility	3.03 (0.95)	1–4
2. Paralinguistic aspects	2.96 (0.55)	2–4
3. Distance in communication	3.32 (1.21)	2–5
4. Physical contact	3.49 (1.32)	2–5
5. Body posture	3.14 (0.13)	3–4
6. Using and performing gestures	3.31 (0.97)	3–5
7. Facial expression	4.11 (0.58)	3–5
8. Gaze	3.24 (0.10)	3–4
9. Use of synonyms	2.70 (0.63)	2–4
10. Number of known and used words	3.23 (1.19)	2–5
11. Interpretation of ambiguous expressions and comments	1.86 (0.75)	1–3
12. Understanding and reacting to irony	1.69 (0.22)	1–2
13. Understanding and reacting to humor	2.48 (0.42)	2–4
14. Word construction	2.71 (0.65)	2–3
15. Appropriate grammatical structure	2.61 (0.61)	2–3
16. Ordered relationship of ideas	2.36 (0.35)	2–3
17. Adapting the communicative style to the context	2.55 (0.48)	2–4
18. Adapting the topics to the conversation	2.48 (0.25)	2–3
19. Subject changes in the conversation	2.51 (0.27)	2–3
20. Maintaining and following the conversation	2.48 (0.19)	2–3
21. Response time to a question	2.74 (0.67)	2–3
22. Interrupting when other interlocutors are speaking	2.53 (0.22)	2–3
23. Quantity of information	2.75 (0.59)	2–3
24. Understanding other people in conversation	2.90 (0.63)	2–3
25. Understanding towards other people in the conversation	3.0 (1.0)	2–4
Mean global score	2.80 (0.91)	1–5

Standard deviations in parentheses

The medium score of each block was calculate and the results were: block I (intelligibility and paralanguage) = 2.99 (0.12), block II (non-verbal communication) = 3.43 (0.65), block III (lexical competence and cohesion) = 2.96 (0.25), block IV (semantic-pragmatic capacities) = 2.01 (0.37), block V (morpho-syntactic characteristics) = 2.56 (0.18), block VI (ability to adapt to the interlocutor and to the communicative situation) = 2.55 (0.48) and block VII (relevance of information) = 2.67 (0.41). The results indicated that significant differences appeared between block IV and blocks I, II and III (*p* < 0.05) and block II and the blocks IV, V and VI (*p* < 0.05).

Regarding the development of the responses offered by family members, it has been observed that items that in the perception about intelligibility of speech, 16% of family members consider that intelligibility is awful in people with WS.

In the item that evaluates suprasegmental aspects such as intonation, rhythm, etc., the results about the perception of families are: 32% positive (excellent or good), 53% regular and 15% negative (bad or very bad). As can be seen, the only item showing a score above 4 (good) is the one referring to facial expression. Most of the items related to non-verbal language (3–8) are between 3 and 4 points. The items that collect the development and the various subareas of oral language (1–2, 9–25) have average scores between 2 and 3 points, which is manifested in the parents’ perception that these aspects are quite abnormal in people with WS (between bad and fair). It should be noted that there were especially low scores in the areas of understanding figurative language with a score of between bad and very bad, interpreting ambiguous expressions and comments (1.86) and understanding and reacting to irony (1.69).

For example, analyzing the item that talks about the proximity that is maintained with the interlocutor in a conversation, the parents have referred very well or well in 38% in WS but we also found scores that refer very poorly or poorly with 21% in WS. Regarding the item that speaks of the use of physical contact in communication situations, better results are concluded in WS with 68% of responses that refer very well or well compared to 12% that consider very badly or badly. Continuing the analysis with the next items, we find the body posture they maintain in a conversation. Better scores are observed in WS with 53% of relatives reporting scores of very good or good. The percentage is similar in the body movements of the arms and hands (56%). The best score was found in facial expression (88%).

As for the items referring to expression, the number of words they know and use in their language in WS is 44% positive (very good or good), 41% regular and 15% negative (bad or very bad). The results don´t improve in word construction (35% positive, 29% regular and 36% negative) and construction of phrases and sentences (24% positive, 44% regular and 32% negative).

To finish, we analyze the last item of this block which includes the interpretation of ambiguous expressions and comments, such as set phrases or metaphors. The results are considered very well or well in 3%. The percentage of answers referring to bad or very bad is 56%. We continue with another block that values understanding and reactions to irony and humor. The irony is considered 3% positive, 18% regular and 79% negative and in the case of humor, the results are slightly better (30% positive, 29% regular and 41% negative).

With regard to the last item (26), which is formulated by means of an open question about parents’ general feelings about the communication of their children with WS and their concern in this regard, the majority of the parents were quite concerned about aspects such as the development of verbal communication, the adaptation of linguistic expression to the context, the understanding of language, turn-taking and specific difficulties when engaging in conversations with their peers and expressing their feelings. All these aspects occur continuously and have a negative impact on communication, leading to great concern amongst most families.

## Discussion

The results obtained in this research study have shown that people with WS experience pragmatic difficulties throughout their development from the point of view of their parents, especially concerning those characteristics relating to the use of oral language in communicative contexts. This research confirms that subjects with WS have difficulty following pragmatic rules [34, 46]. Furthermore, these data follow the line of Agüero and Garayzábal [2] in that the pragmatic characteristics related to verbal aspects, such as the relevance and coherence of the discourse, are abnormal. However, the results do not coincide with the above study regarding non-verbal aspects because relative strengths were found in questions related to block I and block II related to intelligibility, paralanguage, and non-verbal communication, such as eye contact, prosody, or voice control, among others. In our case, parents considered these paralinguistic aspects to be the most developed areas in their children with WS. Nor do our results coincide with others suggesting that people with WS make inappropriate use of prosody and pepper their narrations with excessive onomatopoeia and noises [8, 50, 75], even beyond adolescence [35].

Some studies show that in relation to the conversational competence of people with WS, more than 25% of the expressions were inappropriate, which in half of the cases was due to problems of syntax and expressive semantics and an insufficient amount of information provided [61]. Along these lines, the results of our research show that lexical competence does seem to be more preserved (block III), while syntactic competence (block V) seems to show more difficulty compared to the rest of the blocks evaluated.

Regarding discourse comprehension, the results show that the comprehension of figurative and metaphorical language (understanding irony, jokes, metaphors or double meanings) is very poor [19], so the responses belonging to block IV about semantic-pragmatic capacities (understanding of irony and humor, and the interpretation of ambiguous statements) are those that obtain the lowest scores, assuming a weakness for people with Williams Syndrome. It seems clear that people with WS may have more difficulty understanding non-literal language in areas such as sarcasm, metaphor, and simile when compared to people with typical development of the same chronological age [22]. These same authors point out that these differences disappear between people with WS, and people matched for mental age. These results suggest that these difficulties could be related to intellectual disability and, in this case, indicate that the linguistic and cognitive systems on which the understanding of non-literal language is based interact and integrate differently in individuals with WS compared to those with typical development. Furthermore, according to the authors, the difficulties in understanding sarcasm observed in people with WS could be because sarcasm demands more executive functions, such as cognitive flexibility and context integration.

Concerning the ability to adapt to the interlocutor and the communicative situation, the families assign a low to regular score. It seems, therefore, that people with WS also have difficulties in narratives; when explaining the plot, they introduce irrelevant personal experiences and these tend to lack coherence and cohesion [34, 36, 51, 55, 62]. The parents’ perception also coincides with research confirming that difficulties in turn-taking and following the topic of conversation are distinctive features in this group [16, 41, 54, 65, 74].

In addition, we must be aware that these characteristics do not occur in isolation, as difficulties in understanding the opinions of others [29, 64]—and even in maintaining and establishing linguistic relationships with other interlocutors—lead to personal withdrawal. As a result, people with WS tend not to want to participate in social interactions with their peers [11, 63, 68]. In addition, these characteristics of pragmatic competence in people with WS seem to be very similar to those presented by people with autism spectrum disorder [9, 52]. Hence, individuals with Williams Syndrome appear to exhibit limited linguistic variability within their areas of expertise. Moreover, their conversations tend to revolve around subjects they are familiar with and proficient in, often influencing challenges in social interactions.

Regarding the limitations of the study, it is interesting to highlight, on the one hand, that the individual variability of people with WS within the syndrome must be taken into account, and on the other hand, that the ages of people with WS are so diverse and therefore their language development may be dependent on their age. On the other hand, although there are more and more studies that include language development questionnaires completed by parents in different population [1, 5, 26, 27], it would be interesting to complement our data with those provided by professionals working with people with WS on a daily basis.

In conclusion, if we wish to improve the communication skills of people with WS, we should continue insisting that linguistic work and intervention take place throughout all stages of their development, given the pragmatic language difficulties they experience. The pragmatic profile in people with Williams Syndrome continues to present alterations even in adulthood [14]. So, pragmatic intervention can be effective in SW does take advantage of the elements related to the retention and verbalization of the events of the narrative and thus lay the foundation for the organization and understanding of the discourse [12]. However and to complement this work, following García-Medall and Arranz-López [21] and Sotillo et al. [58], future research efforts on the relationship between the mental and linguistic skills of people with WS should continue to be undertaken, with a view to improving their pragmatic competence and, as a result, their relationship with other interlocutors.

### Supplementary Information

**Additional file 1**. Items which are evaluated in the test PAQ.

## Data Availability

The data that support the findings of this study are available from the research team, but restrictions apply to the availability of these data, which were used under licence for the current study and so are not publicly available. The data are, however, available from the authors upon reasonable request.

## Abbreviations

ASWE: Williams syndrome association of Spain
PAQ: Pragmatic awareness questionnaire
WS: Williams syndrome
