# Supplementary material for: Pragmatic skills in people with Williams syndrome: the perception of families
Source: Orphanet J Rare Dis. 2024 Mar 1;19:95. doi: 10.1186/s13023-024-03016-0 (PMC10908058; doi:10.1186/s13023-024-03016-0)
Supplement: Supplementary file 1 — Additional file 1. Items which are evaluated in the test PAQ. [file 13023_2024_3016_MOESM1_ESM.docx]

**Annex 1. Awareness Questionnaire Pragmatics (CCP) (Rodríguez, 2012)**

The questions we pose below will serve as basis for the development of a psycholinguistic exploration work that aims to delve deeper into the behavior communicative and the social use of language. We guarantee the anonymity of the participants and, above all, we appreciate your collaboration in this project.

Age:

Sex:

Evaluate the following items by marking with a cross (X) the most appropriate answer with a score of 1 to 5:

1 = very bad, 2 = bad, 3 = fair, 4 = good, 5 = very good.

1. When she/he talks to someone in contexts normal communication, how do you usually understand that person the message you do you want to transmit to him?
2. In general, you consider that her/his intonation, rhythm, pauses and volume of the voice when speaking are. . .
3. How do you think she/he regulates the physical proximity with the interlocutor in an expensive conversation to face?
4. Value the use of physical contact with the interlocutor in situations of communication that requires it. For example, if she/he huges or shakes hands in the greetings, farewells or when congratulating someone.
5. Assess the body posture when she/he holds a conversation, that is, the disposition that adopt the parts of her/his body. For example, when the interlocutor is sitting, she/he maintains a position body similar to that of this or, for the contrary, she/he is up, walking or in another position.
6. The foot and leg movements that she/he performs while speaking are to him. . .
7. The movements of arms and hands that she/he does while talking they seem to you. . .
8. In general, how do you think the gestures are that accompany the verbal communication? Judge them based on their naturalness.
9. How is her/his expression facial in interactions? For example, the reflective face when they give you a good or a bad bad news, smiles when something turns out funny or pleasant, adopts an expression negative when angry or offended, etc.
10. I think that her/his gaze towards the interlocutors in the conversation is….
11. Assess his/her ability to use different ways of referring to the same thing, for example, through synonyms (instead of repeating the same word, use a different).
12. How do you judge the number of words she/he knows and use the language?
13. Normally, the way of interpreting ambiguous expressions and comments that others do (for example, when they use words with double meanings, metaphors or phrases of the type "raise your head", "don't give up" or have "iron health") result. . .
14. The understanding and reactions to the irony (for example, if a teacher says to a student " good answers!" when in fact it has taken a bad exam) it seems to you that are. . .
15. The understanding and reactions to the humor (for example, when they tell her/him a joke) often seem to you. . .
16. In general, how do you consider the construction of words? For example, consider if she/he uses properly the endings of nouns and verbs, respecting gender concordances, number and time.
17. How do you estimate the construction of phrases and sentences? Judge whether the statements have a structure with proper grammar.
18. In general, what do you think of her/ his way of relate and connect ideas with others when she/he explains or tells something? For example, it usually structures the information following a structure logic or, on the other hand, considers that its ideas appear messy
19. The style or registration changes and the degree of adaptation to the situation communicative and to her/his interlocutor turno ut (when talk to a friend, to a stranger, in a formal or informal context)
20. What extent do the themes that they develop the treaties in a conversation?
21. Rate the degree of acceptance or success that have the theme changes that they suggests or proposes in the conversations.
22. In general, you think that her/his maintenance and monitoring the conversation is. . .
23. The time taken to respond is… when they ask her/ him something she/he appreciates it, especially value the agility with which their participations are developed.
24. How do you rate the interruptions she/he makes to the interlocutor when speaking? For example, intervenes before your interlocutor finish talking or, however, wait until it ends so then you can intervene.
25. Assess the relevance of her/his interventions in a conversation, mainly the degree to which it is relationed to the case
26. In relation to the amount of information you provide to communicate, believes that habitually is….
